# Pheochromocytomas and secreting paragangliomas

**DOI:** 10.1186/1750-1172-1-49

**Published:** 2006-12-08

**Authors:** Pierre-François Plouin, Anne-Paule Gimenez-Roqueplo

**Affiliations:** 1 Hypertension Unit, Hôpital Européen Georges Pompidou, Assistance Publique-Hôpitaux de Paris; Université René Descartes-Paris 5, France; and the COMETE (COrtico and MEdullo-surrenale: les Tumeurs Endocrines) and PGL.NET networks; 2 Department of Genetics, Hôpital Européen Georges Pompidou, Assistance Publique-Hôpitaux de Paris; Université René Descartes-Paris 5, France; and the COMETE (COrtico and MEdullo-surrenale: les Tumeurs Endocrines) and PGL.NET networks

## Abstract

Catecholamine-producing tumors may arise in the adrenal medulla (pheochromocytomas) or in extraadrenal chromaffin cells (secreting paragangliomas). Their prevalence is about 0.1% in patients with hypertension and 4% in patients with a fortuitously discovered adrenal mass. An increase in the production of catecholamines causes symptoms (mainly headaches, palpitations and excess sweating) and signs (mainly hypertension, weight loss and diabetes) reflecting the effects of epinephrine and norepinephrine on **α**- and **β**-adrenergic receptors. Catecholamine-producing tumors mimic paroxysmal conditions with hypertension and/or cardiac rhythm disorders, including panic attacks, in which sympathetic activation linked to anxiety reproduces the same signs and symptoms. These tumors may be sporadic or part of any of several genetic diseases: familial pheochromocytoma-paraganglioma syndromes, multiple endocrine neoplasia type 2, neurofibromatosis 1 and von Hippel-Lindau disease. Familial cases are diagnosed earlier and are more frequently bilateral and recurring than sporadic cases. The most specific and sensitive diagnostic test for the tumor is the determination of plasma or urinary metanephrines. The tumor can be located by computed tomography, magnetic resonance imaging and metaiodobenzylguanidine scintigraphy. Treatment requires resection of the tumor, generally by laparoscopic surgery. About 10% of tumors are malignant either at first operation or during follow-up, malignancy being diagnosed by the presence of lymph node, visceral or bone metastases. Recurrences and malignancy are more frequent in cases with large or extraadrenal tumors. Patients, especially those with familial or extraadrenal tumors, should be followed-up indefinitely.

## Definition, disease name and synonyms

Pheochromocytomas (PH) are neoplasms of chromaffin tissue which synthesize catecholamines. Most of these tumors appear in the adrenal medulla. Ten percent of catecholamine-producing tumors arise from extraadrenal chromaffin tissue and are called extraadrenal PH or secreting paragangliomas (PGL). In decreasing order of frequency, secreting PGL may develop in the Zuckerkandl body, a vestigial chromaffin ganglion located at the root of the upper mesenteric artery, in the sympathetic plexus of the urinary bladder, the kidneys and the heart, or in sympathetic ganglia in the mediastinum, the head or the neck. Most head and neck PGL are non-secreting. Patients with von Hippel Lindau (VHL) disease or familial PGL (see below) may have uni- or bilateral PH, or PH plus secreting or non-secreting PGL [1,2].

## Diagnostic criteria

PH and secreting PGL are defined by the synthesis and/or secretion of catecholamines: dopamine, norepinephrine and/or epinephrine. Catecholamines are partly or totally converted within the tumor by catechol-O-methyltransferase into inactive metabolites, metanephrine and normetanephrine. Consequently, the release of active catecholamines into the circulation may be modest, absent or paroxysmal. The presence of a catecholamine-producing tumor is nevertheless established by the presence of high concentrations of metanephrine or normetanephrine in the plasma or in the urine [2,3].

## Epidemiology

The prevalence of diagnosed cases of PH and PGL in patients with hypertension and in those with adrenal incidentalomas is about 1 per 1,000 [2] and 4% [4], respectively. The incidence in the general population is estimated to be 1 per 100,000 persons per year or less [1]. The whole-life incidence of PH and PGL is high in familial syndromes with these tumors: 1–5% in neurofibromatosis type 1 (NF1), 15–20% in VHL, 30–50% in multiple endocrine neoplasia type 2 (MEN-2) [2], and probably more than 50% in *SDHB *and *SDHD *gene mutation carriers [5,6].

## Clinical description

The increased production of catecholamines by PH and secreting PGL causes symptoms (mainly headaches, palpitations and excess sweating) and signs (mainly hypertension, weight loss and diabetes) that reflect the effects of catecholamines on **α**- and **β**-adrenergic receptors. Signs and symptoms are variable and frequently paroxysmal due to the variable and disorderly release of catecholamines by the tumor. The typical presentation is a combination of variable hypertension with paroxysmal symptoms, either occurring spontaneously or provoked by abdominal hyperpression during anteflexion, micturition or defecation [3].

Diagnosis of PH/PGL may be delayed for several reasons. First, these tumors are rare. Second, hypertension may be absent for long periods as active catecholamines can be converted into biologically inactive metanephrines within the tumor [1,3]. Third, the symptoms and signs are non-specific, and common to both tumoral (in PH/PGL) and neuronal (during stress) release of catecholamines. This explains why the average time lag from the onset of hypertension when present to the diagnosis of the tumor is three years. Indeed, the tumor is often discovered fortuitously during diagnostic testing for symptoms or clinical conditions not related to adrenal disease. Presymptomatic diagnosis during the exploration of incidentally discovered adrenal masses, the so-called incidentalomas, currently accounts for 25% of all cases [7]. Presymptomatic diagnosis is also possible in patients with phenotypic evidence or a family history of a genetic disease that is associated with PH/PGL (see below).

## Etiology

The etiology of tumorigenesis in PH and secreting PGL is unknown, although valuable information has recently been provided by work on the genomics of familial diseases including these tumors. Normal regulation of the RET protein, a tyrosine kinase receptor, is essential for the development of sympathetic neurons. The constitutive activation of *RET *by germ-line (in MEN-2) or somatic (in a minority of sporadic PH) mutations activates various signaling pathways involved in the development of human neuroendocrine tumors including PH [8]. Functional studies of succinate dehydrogenase (SDH)- or mitochondrial complex II-dysfunction in familial PGL have shown a link between carcinogenesis and alterations in SDH subunits B and D. In hereditary cases with germline *SDHD *or *SDHB *mutations, the tumors exhibit loss of the remaining wild-type allele with complete inhibition of complex II electron transfer activity, leading to activation of pro-angiogenic hypoxic-inducible transcription factors (HIFs) [9-11]. The VHL tumor suppressor protein (pVHL) also interacts with HIF after post-translational prolyl-hydroxylation. In the absence of pVHL, HIF becomes stabilized and is free to induce the expression of several target genes that regulate angiogenesis and cell growth [12]. When SDH is inhibited, the accumulation of succinate seems to inhibit the prolyl-hydroxylation of HIF and induce, as in VHL disease, an inappropriate stabilization of HIF [13].

The hypertension associated with PH is primarily due to increased total peripheral resistance. The mechanism of this increase in resistance would seem to be straightforward because noradrenaline, secreted by the majority of PH, is a potent vasoconstrictor through stimulation of **α**-adrenergic receptors. However, when plasma catecholamines are measured in subjects with PH, the correlation between blood pressure (BP) and circulating noradrenaline levels is poor. Several explanations for this poor correlation have been proposed, including the simultaneous release of noradrenaline and of the vasodilator dopamine, variations in the inactivation of adrenaline and noradrenaline by conjugation, the down-regulation of α-adrenergic receptors, and, in some patients with PH, the concomitant release by the tumor of neuropeptide Y and catecholamines [1,2].

## Diagnostic methods

Biochemical investigation for PH/PGL is generally confined to hypertensive patients reporting bouts of headaches, palpitations and sweating, those with hypertension resistant to treatment, and those with incidentalomas or with a familial disease that predisposes to PH and/or PGL. Positive diagnosis of PH and secreting PGL is chiefly based on the quantification of plasma or urinary metanephrines, as this test is more sensitive than both the quantification of urinary vanillyl-mandelate acid or plasma catecholamines [2,3,14-16], and plasma neuropeptide Y or chromogranin A determinations [17,18]. The determination of fractionated catecholamines (epinephrine and norepinephrine) or catecholamine metabolites (metanephrine and normetanephrine) increases diagnostic sensitivity [15] and provides a prognostic indicator: a low ratio of epinephrine to total catecholamines or of metanephrine proper to total metanephrines is associated with immature tumors, particularly extraadrenal or malignant tumors [19,20]. Patients undergoing biochemical tests for PH/PGL should be given instructions to obtain an accurate 24-hour collection of acidified urines (for urinary tests) and to avoid tricyclic antidepressants and paracetamol for 5 days as these medications induce false positive results in plasma metanephrine tests [3]. With accurate plasma and urinary assays readily available, there is little need to subject patients to the hazards of pharmacological provocative tests. Paroxysmal conditions with hypertension and/or cardiac rhythm disorders mimic catecholamine-secreting tumors, particularly panic attacks in which sympathetic activation linked to anxiety reproduces the signs and symptoms of PH. Plasma and urinary metanephrine concentrations are usually normal [2] and the clonidine suppression test is a safe and effective means of excluding a tumor in these conditions.

The purpose of pre-operative imaging tests is to locate the tumor, ascertain whether it is single or multiple, adrenal or ectopic, benign or malignant, and isolated or present with other neoplasms in the context of familial syndromes. Of 192 patients referred for the preoperative work-up of a first PH or PGL, 20 (10%) had bilateral PH and 25 had PGL (13%). Eleven PH and three PGL were malignant (7% of all tumors). Tumors arose in the context of a familial disease in 34 patients (18%), most of whom had associated non-chromaffin tumors [7]. The combination of anatomical imaging studies using computed tomography (CT) or magnetic resonance imaging (MRI) and functional imaging studies (nuclear medicine) yields a sensitivity of nearly 100% for diagnosing catecholamine-producing tumors [21]. CT is the most commonly used anatomical imaging technique but MRI is preferred for children and pregnant patients. Functional imaging using [^123^I] metaiodobenzylguanidine (MIBG) should be performed where available, [^131^I]MIBG scintigraphy having a lower sensitivity. Labetalol and antipsychotic drugs should be withdrawn for several days before the investigations as they reduce [^123^I]MIBG and [^131^I]MIBG uptake. Where these imaging methods give negative (mostly in non-secreting PGL) or discordant results, the investigation should be completed by scintigraphy with nonspecific ligands such as somatostatin receptor scintigraphy or [^18^F]fluorodeoxyglucose PET, fluoro-DOPA or fluoro-dopamine PET. In addition to the primary tumor, CT, MRI or scintigraphy may disclose lymph node, bone, liver or pulmonary metastases, thereby establishing the presence of a malignant PH or PGL.

## Differential diagnosis

Catecholamine-secreting tumors mimic paroxysmal conditions with hypertension and/or cardiac rhythm disorders, particularly panic attacks in which sympathetic activation linked to anxiety reproduces the signs and symptoms of PH. Plasma and urinary metanephrine concentrations are usually normal in these conditions [2]. Acute cardiovascular events, such as myocardial infarction, pulmonary edema and stroke, also induce an increase in catecholamine levels that may be sustained for several days and associated with a rise in plasma or urinary metanephrine concentration. The diagnosis of PH or secreting PGL is excluded in these cases by the normalization of metanephrines ten days after the onset of the event [14]. Adrenomedullary hyperplasia, sometimes present in patients with MEN-2, is distinguished from PH proper by the absence of tumor. Non-secreting PGL can be differentiated from secreting PGL by normal concentrations of catecholamines and metanephrines in the plasma and urine. Patients with hereditary PGL or with VHL disease may harbor both secreting and non-secreting PGL, making the differential diagnosis of the individual tumor difficult.

## Genetic counseling

Prior to 2000, three different familial and syndromic diseases were known to result in PGL and/or PH: MEN-2 induced by germline inactivating mutations in the *RET *proto-oncogene; VHL disease due to mutations in the tumor suppressor gene *VHL*; and NF1 caused by mutations in the *NF1 *gene. More recently, the identification of mutations in the *SDH *(*SDHD*, *SDHB*, *SDHC*) genes in hereditary PGL and PH [22] has led to changes in the genetic counseling and work-up for affected patients [5,23-25].

**Figure 1:**
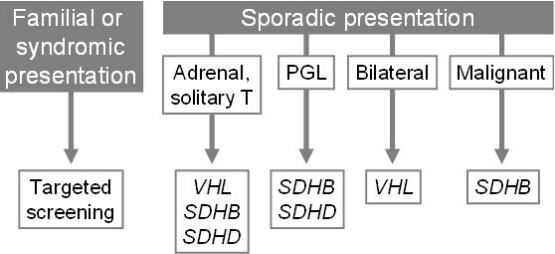
Genetic testing in patients with pheochromocytomas and secreting paragangliomas (Adapted from [24]).

Genetic testing is indicated for all patients with PGL and/or PH, whatever the location of the tumor and the age of the subjects, but such testing may also be indicated by clinical and familial features (Figure 1). Targeted genetic testing should be proposed to patients with phenotypic clues and/or familial history for MEN-2 (medullary thyroid cancer, hyperparathyroidism), VHL disease (hemangioblastoma, renal or pancreatic tumors), or hereditary PGL (head and neck PGL, familial history in the paternal branch). The family pedigree may also direct the genetic testing. The disease displays autosomal dominant inheritance for all PH susceptibility genes but in families with *SDHD *mutations the disease is transmitted exclusively *via *the fathers due to genomic maternal imprinting. In patients with an apparently sporadic presentation, the *VHL *and *SDHB *genes should be analyzed in first. In carriers of *SDHB *mutations, the resulting tumor is usually a secreting or non-secreting PGL, which may be malignant [4,24,26]. The identification of a causative mutation in one affected patient should lead to presymptomatic genetic testing of the family because the early detection of small tumors in individuals determined to be at risk would reduce the morbidity of the disease. Presymptomatic genetic testing should be offered to all first-degree relatives if a causative mutation is detected in the index case.

## Management

### Pre-operative management

BP should be normalized whenever possible before surgery because the incidence of perioperative complications is consistently and independently linked to preoperative BP [27]. The control of BP requires **α**- and **β**-adrenergic antagonists. As the majority of PH and PGL secrete predominantly the **α**-agonist norepinephrine, **α**-adrenergic antagonists are the cornerstone of hypertensive control. Non-competitive **α**-blockers such as phenoxybenzamine bind covalently to **α**-receptors producing an irreversible blockade and a reflex tachycardia due to the inhibition of presynaptic **α**_2_-adrenergic receptors. They are therefore useful for prolonged antihypertensive treatment of malignant PH/PGL but should be avoided pre-operatively because they increase the risk of acute hypotension during tumor removal and the immediate post-operative period. Selective **α**_1_-blockers such as prazosine or its derivative doxazosine are more suitable because they have a shorter duration of action and do not produce **α**_2_-anti-adrenergic-mediated tachycardia. The first dose of prazosine may induce a sharp drop in BP, and therefore the dose should be increased progressively from 0.5 to 5 mg tid. Alpha-adrenergic blockade generally gives rise to tachycardia, secondary to catecholamine **β**-receptor stimulation. This requires the subsequent addition of a **β**-blocker. When adrenergic blockade is insufficient, antihypertensive therapy may be completed by administering a dihydropyridine and/or an angiotensin-converting enzyme inhibitor. In view of the variability of BP in PH, it may be useful to document the response to antihypertensive treatment with a 24-hour ambulatory BP monitor. Prevention of arrhythmias is based on **β**-blockade and careful correction of hypokalemia: PH causes a state of secondary hyperaldosteronism, resulting in increased potassium loss.

### Anesthesia and surgical management

Large variations in BP and cardiac rhythm may be expected during the anesthetic management of patients with PH, particularly during induction, intubation, peritoneal incision, and tumor handling and devascularization. Radial artery pressure, pulmonary artery pressure through a Swan-Ganz catheter, and electrocardiogram (ECG) should be monitored continuously. Infusions of short-acting vasodilator or anti-arrhythmic agents should be prepared in advance and begun as soon as necessary. Laparoscopic surgery has supplanted the use of open surgery in the management of most PH and intraabdominal PGL. Adrenocortical-sparing surgery may be performed using laparoscopy in patients with hereditary forms of PH [28].

### Post-operative and long term follow-up

Biochemical evidence for the cure of the tumor cannot be obtained immediately: the output of catecholamine metabolites remains high, albeit decreasing, during the first week following tumor resection. This output represents the emptying of extratumoral pools of catecholamines and should not be interpreted as only partial response to surgery. The normalization of plasma or urinary metanephrine concentration should be checked 10 days after surgery. If the metanephrine concentrations remain high, [^123^I]MIBG scintigraphy should be performed and may disclose distant metastases whose MIBG uptake was masked pre-operatively by the primary tumor's higher metabolic activity.

As PH and PGL can recur, patients operated for PH/PGL should have a life-long follow-up, the minimum annual checks including BP measurement and plasma or urinary metanephrine determination. In a cohort of patients operated upon for PH or PGL, the 10-year probability of recurrence (defined as the reappearance of the disease after eradication of the tumor had been confirmed by negative biochemical and imaging tests) was 16% [7]. Patients with recurrences were younger, had larger tumors, and were more likely to have familial disease and/or bilateral, or extra-adrenal PGL than patients with no recurrence. Recurrences were malignant in one patient out of two.

Malignant PH/PGL is compatible with prolonged survival with symptom-free intervals varying from months to decades. Factors associated with a longer survival seem to be an early diagnosis and excision of the primary tumor, and, whenever possible, aggressive excision of any recurrence or soft-tissue metastases. When recurrences are small with an accessible vascular pedicle, surgical excision may be preceded, or replaced, by therapeutic embolization. In cases where soft-tissue or skeletal metastases are too widespread to make surgery or embolization feasible, several palliative therapies may be considered. Pharmacologic treatment aimed at the long-term blockade of catecholamine synthesis with **α**-methyl-p-tyrosine may allow a good quality of life but does not affect tumor progression. Conventional radiotherapy may provide good palliation in cases of painful metastases. Metabolic radiotherapy with [^131^I]MIBG and chemotherapy may provide clinical, hormonal and sometimes tumoral improvement [29]. Such therapies have only been evaluated in limited uncontrolled trials and their effect on survival is not known. They should therefore only be considered after the surgical excision of primary or recurrent tumors.

## Unresolved questions

Current research aims to unravel the etiology of sporadic PH and PGL; to develop tumor-mass markers for PGL with little or no catecholamine synthesis; to assess genotype-phenotype relationships and disease penetrance in rare familial diseases; and to design trials of adjuvant therapies or alternatives to surgery for aggressive tumors.

**Figure 2:**
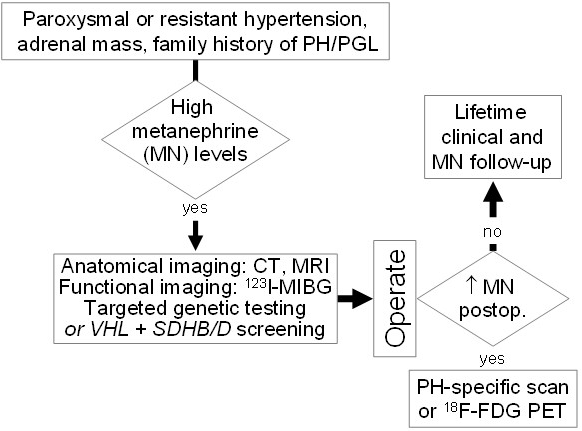
Algorithm for the initial management and long term follow-up in patients with pheochromocytomas and secreting paragangliomas.

## References

[B1] Bravo EL, Tagle R (2003). Pheochromocytoma: state-of-the-art and future prospects. Endocr Rev.

[B2] Pacak K, Linehan WM, Eisenhofer G, Walther MM, Goldstein DS (2001). Recent advances in genetics, diagnosis, localization, and treatment of pheochromocytoma. Ann Intern Med.

[B3] Eisenhofer G, Goldstein DS, Walther MM, Friberg P, Lenders JW, Keiser HR, Pacak K (2003). Biochemical diagnosis of pheochromocytoma: how to distinguish true- from false-positive test results. J Clin Endocrinol Metab.

[B4] Mantero F, Terzolo M, Arnaldi G, Osella G, Masini AM, Ali A, Giovagnetti M, Opocher G, Angeli A (2000). A survey on adrenal incidentaloma in Italy. Study Group on Adrenal Tumors of the Italian Society of Endocrinology. J Clin Endocrinol Metab.

[B5] Neumann HP, Pawlu C, Peczkowska M, Bausch B, McWhinney SR, Muresan M, Buchta M, Franke G, Klisch J, Bley TA, Hoegerle S, Boedeker CC, Opocher G, Schipper J, Januszewicz A, Eng C, European-American Paraganglioma Study Group (2004). Distinct clinical features of paraganglioma syndromes associated with SDHB and SDHD gene mutations. JAMA.

[B6] Benn DE, Gimenez-Roqueplo AP, Reilly JR, Bertherat J, Burgess J, Byth K, Croxson M, Dahia PL, Elston M, Gimm O, Henley D, Herman P, Murday V, Niccoli-Sire P, Pasieka JL, Rohmer V, Tucker K, Jeunemaitre X, Marsh DJ, Plouin PF, Robinson BG (2006). Clinical presentation and penetrance of pheochromocytoma/paraganglioma syndromes. J Clin Endocrinol Metab.

[B7] Amar L, Servais A, Gimenez-Roqueplo AP, Zinzindohoue F, Chatellier G, Plouin PF (2005). Year of diagnosis, features at presentation, and risk of recurrence in patients with pheochromocytoma or secreting paraganglioma. J Clin Endocrinol Metab.

[B8] Ichihara M, Murakumo Y, Takahashi M (2004). RET and neuroendocrine tumors. Cancer Lett.

[B9] Gimenez-Roqueplo AP, Favier J, Rustin P, Mourad JJ, Plouin PF, Corvol P, Rotig A, Jeunemaitre X (2001). The R22X mutation of the SDHD gene in hereditary paraganglioma abolishes the enzymatic activity of complex II in the mitochondrial respiratory chain and activates the hypoxia pathway. Am J Hum Genet.

[B10] Gimenez-Roqueplo AP, Favier J, Rustin P, Rieubland C, Kerlan V, Plouin PF, Rotig A, Jeunemaitre X (2002). Functional consequences of a SDHB gene mutation in an apparently sporadic pheochromocytoma. J Clin Endocrinol Metab.

[B11] Favier J, Plouin PF, Corvol P, Gasc JM (2002). Angiogenesis and vascular architecture in pheochromocytomas: distinctive traits in malignant tumors. Am J Pathol.

[B12] Kim WY, Kaelin WG (2004). Role of VHL gene mutation in human cancer. J Clin Oncol.

[B13] Selak MA, Armour SM, MacKenzie ED, Boulahbel H, Watson DG, Mansfield KD, Pan Y, Celeste Simon M, Thompson CB, Gottlieb E (2005). Succinate links TCA cycle dysfunction to oncogenesis by inhibiting HIF-alpha prolyl hydroxylase. Cancer Cell.

[B14] Héron E, Chatellier G, Billaud E, Foos E, Plouin PF (1996). The urinary metanephrine to creatinine ratio in the diagnosis of pheochromocytoma. Ann Intern Med.

[B15] Lenders JW, Pacak K, Walther MM, Linehan WM, Mannelli M, Friberg P, Keiser HR, Goldstein DS, Eisenhofer G (2002). Biochemical diagnosis of pheochromocytoma: which test is best?. JAMA.

[B16] Kudva YC, Sawka AM, Young WF (2003). The laboratory diagnosis of adrenal pheochromocytoma: the Mayo Clinic experience. J Clin Endocrinol Metab.

[B17] Hsiao RJ, Parmer RJ, Takiyyuddin MA, O'Connor DT (1991). Chromogranin A storage and secretion: sensitivity and specificity for the diagnosis of pheochromocytoma. Medicine (Baltimore).

[B18] Des Senanayake P, Denker J, Bravo EL, Graham RM (1995). Production, characterization, and expression of neuropeptide Y by human pheochromocytoma. J Clin Invest.

[B19] Plouin PF, Chatellier G, Fofol I, Corvol P (1997). Tumor recurrence and hypertension persistence after successful pheochromocytoma operation. Hypertension.

[B20] van der Harst E, de Herder WW, de Krijger RR, Bruining HA, Bonjer HJ, Lamberts SW, van den Meiracker AH, Stijnen TH, Boomsma F (2002). The value of plasma markers for the clinical behaviour of phaeochromocytomas. Eur J Endocrinol.

[B21] Ilias I, Pacak K (2004). Current approaches and recommended algorithm for the diagnostic localization of pheochromocytoma. J Clin Endocrinol Metab.

[B22] Baysal BE (2003). On the association of succinate dehydrogenase mutations with hereditary paraganglioma. Trends Endocrinol Metab.

[B23] Neumann HP, Bausch B, McWhinney SR, Bender BU, Gimm O, Franke G, Schipper J, Klisch J, Altehoefer C, Zerres K, Januszewicz A, Eng C, Smith WM, Munk R, Manz T, Glaesker S, Apel TW, Treier M, Reineke M, Walz MK, Hoang-Vu C, Brauckhoff M, Klein-Franke A, Klose P, Schmidt H, Maier-Woelfle M, Peczkowska M, Szmigielski C, Eng C, Freiburg-Warsaw-Columbus Pheochromocytoma Study Group (2002). Germ-line mutations in nonsyndromic pheochromocytoma. N Engl J Med.

[B24] Amar L, Bertherat J, Baudin E, Ajzenberg C, Bressac de Paillerets B, Chabre O, Chamontin B, Delemer B, Giraud S, Murat A, Niccoli-Sire P, Richard S, Rohmer V, Sadoul JL, Strompf L, Schlumberger M, Bertagna X, Plouin PF, Jeunemaitre X, Gimenez-Roqueplo AP (2005). Genetic testing in pheochromocytoma or functional paraganglioma. J Clin Oncol.

[B25] Plouin PF, Gimenez-Roqueplo AP (2006). The genetic basis of pheochromocytoma: who to screen and how?. Nat Clin Pract Endocrinol Metab.

[B26] Gimenez-Roqueplo AP, Favier J, Rustin P, Rieubland C, Crespin M, Nau V, Khau Van Kien P, Corvol P, Plouin PF, Jeunemaitre X, COMETE Network (2003). Mutations in the SDHB gene are associated with extra-adrenal and/or malignant phaeochromocytomas. Cancer Res.

[B27] Plouin PF, Duclos JM, Soppelsa F, Boublil G, Chatellier G (2001). Factors associated with perioperative morbidity and mortality in patients with pheochromocytoma: analysis of 165 operations at a single center. J Clin Endocrinol Metab.

[B28] Walther MM (2002). New therapeutic and surgical approaches for sporadic and hereditary pheochromocytoma. Ann N Y Acad Sci.

[B29] Eisenhofer G, Bornstein SR, Brouwers FM, Cheung NK, Dahia PL, de Krijger RR, Giordano TJ, Greene LA, Goldstein DS, Lehnert H, Manger WM, Maris JM, Neumann HP, Pacak K, Shulkin BL, Smith DI, Tischler AS, Young WF (2004). Malignant pheochromocytoma: current status and initiatives for future progress. Endocr Relat Cancer.

